# Combining ability and heterosis studies for grain iron and zinc concentrations in pearl millet [*Cenchrus americanus* (L). Morrone]

**DOI:** 10.3389/fpls.2022.1029436

**Published:** 2023-01-25

**Authors:** R. Thribhuvan, S. P. Singh, Mukesh S. Sankar, Anju M. Singh, M. Mallik, Tripti Singhal, Jitendra Kumar Meena, C. Tara Satyavathi

**Affiliations:** ^1^ Division of Genetics, ICAR- Indian Agricultural Research Institute, New Delhi, India; ^2^ Project Co-ordinator, ICAR- Central Research Institute for Jute and Allied Fibres, Barrackpore, India; ^3^ ICAR- All India Coordinated Research Project on Pearl Millet, Jodhpur, India

**Keywords:** combining ability, grain iron and zinc density, gene action, heterosis, L × T mating design, pearl millet

## Abstract

Iron (Fe) and zinc (Zn) deficiency has been identified as a major food-related health issue, affecting two billion people globally. Efforts to enhance the Fe and Zn content in food grains through plant breeding are an economic and sustainable solution to combat micronutrient deficiency in resource-poor populace of Asia and Africa. Pearl millet, *Cenchrus americanus* (L). Morrone, considered as a hardy nutri-cereal, is the major food crop for millions of people of these nations. As an effort to enhance its grain mineral content, an investigation was conducted using line × tester analysis to generate information on the extent of heterosis, gene action, combining ability for grain yield potential, and grain mineral nutrients (Fe and Zn). The partitioning of variance attributable to parents indicated that the lines and testers differed significantly for the traits studied. For most of the attributes, hybrids that were superior to the parents in the desired direction in terms of *per se* performance were identified. The analysis of combining ability variance indicated the preponderance of both additive and non-additive genetic effects. Thus, reciprocal recurrent selection can be used to develop a population with high–grain Fe and Zn contents. The Fe and Zn content in grain exhibited a highly significant and positive association between them, whereas the Fe and Zn contents individually showed a negative, albeit weak, correlation with grain yield and a moderate positive relation with grain weight. This indicates that mineral nutrient contents in grains can be improved without significant compromise on yield. The consistency of these trends across the environment suggests that these findings could be directly used as guiding principles for the genetic enhancement of Fe and Zn grain content in pearl millet.

## 1 Introduction

Micronutrient malnutrition or hidden hunger results from the deficiency of one or more micronutrients. Globally, about two-thirds of the population is reported to be suffering from this hidden hunger due to the dearth of essential micronutrients in their diet (Kumar et al., 2018). Cereal grains are the main source of proteins, carbohydrates, and micronutrients in human nutrition. The human body requires about two dozen of essential elements to ensure optimal health. For humans, several beneficial mineral elements such as iron (Fe), zinc (Zn), boron (B), copper (Cu), manganese (Mn), and cobalt (Co) are necessary in trace quantities (Kumar et al., 2015). Of these, Fe and Zn are primarily deficient in human diets (Mahendrakar et al., 2019). Mineral malnutrition is a major diet-related health issue for people in the developing world due to inadequate access to and affordability of horticultural products (fruits and vegetables) and meat. Reliance on carbohydrate dominated cereal grain–based diets further exacerbates the problem. The consequences of malnutrition can be catastrophic, resulting in mental retardation, impaired fitness, low productivity, lethargy, and decreased stamina (Kumar et al., 2016).

Fe and Zn requirements can be fulfilled through several ways. Food fortification and dietary supplementation are commonly adopted strategies to address malnutrition. However, these solutions are impractical and entail a significant expenditure (Jeong and Guerinot, 2008). Crop biofortification *via* plant breeding techniques has a potential to solve the issue of Fe-Zn deficiency in a sustainable and lucrative manner. Biofortification through genetic improvement has resulted in tangible increase in Fe levels of many crops like corn (*Zea mays*), wheat (*Triticum aestivum*), sorghum (*Sorghum bicolor*), pearl millet (*Cenchrus americanus*), and cowpea (*Vigna unguiculata*).

Pearl millet is the most important cereal crops next to rice, wheat, corn, barley, and sorghum with a very high photosynthetic efficiency and dry matter output being a C_4_ species. It can be cultivated in even the most unfavorable agro-ecologies where other cereals, such as rice or wheat, do not thrive and fail to generate economic outputs. It can play a significant role not just contributing to the food and nutritional welfare of the poor in the pearl millet–growing areas of India and Sub-Saharan Africa but can have a potential health benefit for the wealthier as well (Rai et al., 2012). India is the largest producer of pearl millet in the world with an area of 6.93 million ha with an average production of 8.61 million tones and productivity of 1,243 kg/ha during 2018–2019 (Directorate of Millets Development, 2020). It is a rich source of fiber and minerals—notably Fe, Zn, and calcium—thus, rightly said as “nutri-cereal”. Despite these health benefits, it is less popular as a staple due to lack of awareness about its nutritious value, proper processing technologies, and remunerative prices for farmers. This is apparent from the geographical distribution of pearl millet intake across India. About 19% to 63% of the total intake of Fe and 16% to 56% of the total intake of Zn was restricted to the states of Rajasthan, Maharashtra, and Gujarat (Rao et al., 2006). High genetic diversity for Fe and Zn available in breeding lines, improved populations, and germplasm were already reported in pearl millet offers good opportunities for breeding pearl millet varieties with increased levels of these micronutrients (Velu et al., 2007; Velu et al., 2008; Rai et al., 2012).

Combining ability analysis is considered helpful in determining the right combination of parents, which would generate heterotic hybrid and superior segregants in the succeeding generations. Such studies also throw some light on nature and magnitude of the gene action involved in the inheritance of target traits, which further dictate the breeding method to be adopted in segregating generations for trait improvement. There are a number of strategies for determining the breeding value of lines in terms of its combining ability and genetic composition. The line × tester study suggested by Kempthorne (1957) is the widely used method to determine the combining ability of the parent and general combining ability (GCA) and specific combining ability (SCA) effects and variances for crosses of various traits. Bearing this in mind, the present research work was undertaken to elucidate the combining ability of grain minerals and yield components to ascertain the nature and magnitude of gene actions of parental lines and hybrids developed in a line × tester design.

## 2 Materials and methods

### 2.1 Plant materials

Three CMS lines along with 10 restorers (testers) of pearl millet were used for the present study. These three CMS lines were crossed with 10 restorers in line × tester mating design as suggested by Kempthorne (1957) and reviewed by Dabholkar (1992), yielding 30 hybrids in *Kharif* (2018). The details of parents along with their pedigree are given in **Table 1**. Thus, a total of 44 genotypes including 13 parents, 30 crosses, and one check (Pusa1201) constituted the experimental material for combining ability study. The parentals used for hybrid development were of diverse origin with a wide variation for grain Fe and Zn densities, grain yield, and other agronomic traits.

**Table 1:** Origin and parentage of B-lines (maintainer counterparts of A-lines) and restorer lines (R-lines).

Sl no.	Line	Parentage
B lines		
1	ICMB-02555	CMV87901-175-2-3-2-B-1
2	ICMB-07999	(HTBC 48-B-1-1-1-5 × B-bulk)-25-1-B-B
3	ICMB-12222	(ICMB 95444 × ICMB 92111)-4-B-4-3-B-B
R lines		
4	A5RT-17/4	(IPC 107 × ICMV 91059 S1-14-2-1-1-2)-6-2-5-B-B-6-1
5	A5RT-17/5	(IPC 1617 × SDMV 90031-S1-84-1-1-1-1)-28-1-1-2-B-B-2-1-3
6	A5RT-17/6	(IPC 1617 × SDMV 90031-S1-84-1-1-1-1)-28-1-1-2-B-B-2-2-1
7	A5RT-17/7	(IPC 1617 × SDMV 90031-S1-84-1-1-1-1)-28-1-1-2-B-B-2-3-1
8	A5RT-17/8	(IPC 1617 × SDMV 90031-S1-84-1-1-1-1)-28-1-1-2-B-B-2-6-1
9	A5RT-17/16	(IPC 1268 × ICMV 91059 S1-58-2-2-2-1)-7-3-4-1-B-B-B-4-1
10	A5RT-17/19	(IPC 107 × ICMV 91059 S1-14-2-1-1-2)-19-1-2-2-3-B-5-2-1
11	A5RT-17/25	[(IPC 337 × SDMV 90031-S1-84-1-1-1-1) × RCB-2-S1-144-2-2-2-1-1-1]-2-4-2-3-3-B-B-3-1
12	A5RT-17/26	[(IPC 337 × SDMV 90031-S1-84-1-1-1-1) × RCB-2-S1-144-2-2-2-1-1-1]-2-4-2-3-3-B-B-5-4
13	A5RT-17/29	(IPC 107 × SDMV 90031-S1-84-1-1-1-1)-1-1-2-2-B-B-B

### 2.2 Field trials

#### 2.2.1 Crossing

Three A-lines (ICMA-02555, ICMA-07999, and ICMA-12222) were crossed with 10 diverse R-lines at IARI, New Delhi during *Kharif* (2018). Individual healthy plants were tagged and used for making plant × plant crosses to produce the F_1_s. Before stigma emergence both the male and female parents were covered with brown bags. On the day of crossing, bagged panicles of female parents were checked for complete stigma emergence and bagged male parent panicles for pollen shedding. Pollens from target parent were collected between 10:00 am and 11:30 am and were dusted on the female parent’s panicle in which stigma has fully emerged. The pollinated panicle was immediately covered to prevent outcrossing. The details of cross such as parents and date of pollination were mentioned on the tag. Crossed panicles were finally collected at maturity (40 days after pollination) and dried. Then, panicles were threshed to get F_1_ (hybrid) seed.

#### 2.2.2 Evaluation of parents and hybrids

Hybrids and parents were planted during the summer season 2019 (E1) at ICRISAT, Patancheru, and during the rainy season 2019 (E2) at IARI, New Delhi. A replicated (twice) randomized complete block design was followed at both the locations. Each genotype was sown in two rows of 2 m length with a spacing of 75 cm between rows in the rainy season in 2019 and 60 cm in the summer season in 2019. The recommended agronomic practices for healthy crop growth were adopted. Panicles were harvested at maturity from five randomly selected plants, sun-dried to appropriate moisture content (10%–13%) followed by manual threshing for the preparation of grain samples. After taking the grain weight [considered as grain yield per plant (GYPP) in grams], 1000-grain weight were counted from each sample and weighed to estimate 1,000-grain weight. The average value of five plants per each replication was used for downstream analysis. At each step care, it was ensured that grain did not come in contact with metal containers or instruments to generate reliable estimates of micronutrients.

### 2.3 Estimation of grain iron, zinc, and phytate contents

Fe and Zn contents in cleaned grain samples were measured using energy-dispersive x-ray fluorescence (XRF). The XRF machine model X-Supreme 8000 (M/s Oxford Inc., USA) was used for the purpose of following the method by Govindaraj et al. (2016). Phytic acid (phytate) was determined by an assay unique to the measurement of phosphorous release based on available phosphorous from phytate, myo-inositol (phosphate)n, and monophosphate esters by phytase and alkaline phosphatase using the Phytic Acid/Total Phosphorus Assay Kit (Megazyme, 2016).

### 2.4 Molar ratio of phytate/mineral

The molar ratios of Phy/mineral were first determined by converting the quantities of phytic acid, Fe/Zn in mg g^−1^ to mmol g^−1^ using a molecular mass unit (phytate, 660.8 g/mol; Fe, 55.8 g/mol; Zn, 65.38 g/mol). These values were then used to derive corresponding molar ratios.

### 2.5 Statistical analysis

Replication-wise data of all the traits were analyzed using the software Analysis of Genetic Designs with R for Windows (AGD-R) (Rodríguez et al., 2015). Line × tester model for female × male hybrid was executed to generate estimates of the GCA for lines and testers as well as the SCA effects for hybrids. Furthermore, a mixed-effect model was adopted with genotypes (lines, testers, and hybrids) and environments as fixed effects, and interactions (site × line, site × tester, line × tester, and site × line × tester) and replication as random effects. The ANOVA model considered for the combining ability analysis through line × tester design is as follows (Arunachalam, 1974; Dabholkar, 1999):


$$Y_{ijk}=\;\mu +g_{i}+g_{j}+s_{ij}+r_{k}+e_{ijk}$$

where Y_ijk_ is the mean trait value measured on cross made between ith line and jth tester in the kth replication; μ is the grand mean; g_i_ is the GCA effect of the ith parent; g_j_ is the GCA effect of the parent j; s_ij_ is the SCA effect of cross i × j; r_k_ is the replication effect; and e_ijk_ is the environmental effect. Three estimates of heterosis, i.e., mid-parent heterosis (MPH), better-parent heterosis (BPH), and standard heterosis (SH), were calculated for individual environments and the mean of two environments (Hallauer and Miranda, 1981). The tests of significance for MPH and BPH were done *via* “t” test.

## 3 Results

### 3.1 Genetic variability

Analysis of variance for combining ability (**Table 2**) revealed that the variance due to lines and tester was significant for all characters under recorded at both environments and combined over environment except for the grain yield per plant in summer. The source of variance due to line × tester interaction was also significant for all traits in the individual environments and pooled environment except for grain yield per plant in summer season. Variance for the environments was highly significant for all the characters under study except for grain phytate content.

Table 2Mean squares and genetic components for traits studied in line × tester trial of pearl millet in the 2019 summer season (E1) and 2019 rainy season (E2).

**Table:** 

Source of Variation	df^†^	Mean sum of squares											
		Fe (µgg^-1^)			Zn (µgg^-1^)			TGW (g)			GYPP (g)		
		E1	E2	Combined	E1	E2	Combined	E1	E2	Combined	E1	E2	Combined
Environment	1			11688.49**			7160.61**			63.73**			2539.75**
Replications	(1) 2	1.550000	32.08**	16.82*	1.05	2.68	1.86	2.14	0.00	1.07	2.5	180.359**	98.17**
Hybrid	29	339.7**	620.45**	772.12**	171.39**	263.79**	352.51**	1.44**	5.67**	4.73**	10.444	39.897**	35.65**
Lines	(2) 2	334.97**	65.077**	347.33**	136.83**	333.21**	397.76**	0.31	4.49**	1.98**	28.66*	312.922**	252.77**
Testers	(9) 9	708.78**	1360.91**	1945.41**	248.24**	449.51*	636.51**	0.71	2.52**	2.02**	14.214	12.625	19.72*
Lines × Testers	(18) 18	155.62**	311.94**	232.67**	136.81**	163.22**	205.47**	1.94**	7.37**	6.40**	6.535	23.19*	19.48**
Hybrid × Environment	29			187.99**			82.68**			2.38**			14.69*
Line × Environment	2			52.70**			72.28**			2.81**			88.85**
Tester × Environment	9			124.26**			61.24**			1.21**			7.11
Line × Tester × Environment	18			234.88**			94.55**			2.92**			10.24
Error	(29) 58	2.93	6.75	4.84	2.93	1.78	2.47	0.54	0.24	0.4	6.304	9.836	8.26
		**Genetic Components**											
Genotype Variance		168.37	306.85	5597.89	84.11	131.01	2555.66	0.45	2.72	34.31	2.40	14.51	258.46
Line Variance		8.97	0.00	2.87	0.00	8.50	4.81	0.00	0.00	0.00	1.11	14.49	5.83
Tester Variance		92.19	174.83	142.73	18.57	47.72	35.92	0.00	0.00	0.00	1.28	0.00	0.02
LinexTester Variance		76.35	152.59	56.96	66.82	80.72	50.75	0.70	3.57	1.50	0.45	6.15	2.81
Additive Variance		227.11	380.72	332.85	42.68	124.11	90.72	0.00	0.00	0.00	4.82	20.61	9.97
Dominance Variance		305.38	610.37	227.83	267.29	322.88	203.00	2.80	14.27	6.01	1.80	24.62	11.23
Contributions of lines		6.80	0.72	3.10	5.51	8.71	7.79	1.48	5.45	2.89	18.93	54.09	38.65
Contributions of testers		64.76	68.07	78.20	44.95	52.88	56.04	15.25	13.81	13.26	42.24	9.82	26.52
Contributions of lxt		28.44	31.21	18.70	49.54	38.40	36.18	83.27	80.74	83.86	38.84	36.09	34.83

**Table 2. Continued:** 

Source of Variation	df	Mean sum of squares								
		PA (mgg^-1^)			PA/Fe			PA/Zn		
		E1	E2	Combined	E1	E2	Combined	E1	E2	Combined
Environment	1			0.000026			0.025**			0.051**
Replications	(1) 2	0.00000659	0.0002482*	0.00015**	0.0000001	0.0005713**	0.00029**	0.0000028	0.0007966*	0.00046**
Hybrid	29	0.00010438**	0.00021115**	0.00026**	0.0011854**	0.0007508**	0.0016**	0.0024542**	0.0011905**	0.003**
Lines	(2) 2	0.00042237*	0.00050228	0.00092**	0.0001666	0.0001608	0.00032**	0.0031733	0.0029251	0.0061**
Testers	(9) 9	0.000088	0.00023921	0.00026**	0.0024944**	0.0015719**	0.0039**	0.0035351	0.0014439	0.0045**
Lines × Testers	(18) 18	0.00007724**	0.00016477**	0.000193**	0.0006442**	0.0004058**	0.00058**	0.0018339**	0.0008711**	0.0019**
Hybrid × Environment	29			0.000051**			0.00033**			0.00064**
Line × Environment	2			0.000004			0.00000055			0.000007
Tester × Environment	9			0.000066**			0.00014**			0.0005**
Line × Tester × Environment	18			0.000049*			0.00046**			0.00079**
Error	(29) 58	0.0000085	0.0000400	0.000022	0.0000433	0.0000643	0.0000449	0.0000809	0.0001143	0.000084
		**Genetic Components**								
Genotype Variance		4.78E-05	8.77E-05	0.001914	0.00057	0.00035	0.011632702	0.00119	0.00055	0.021752776
Line Variance		1.73E-05	1.69E-05	0.000018	0.00000	0.00000	0	0.00007	0.00010	0.000104488
Tester Variance		1.79E-06	1.24E-05	0.000006	0.00031	0.00019	0.000278941	0.00028	0.00010	0.000213954
LinexTester Variance		3.42E-05	6.45E-05	0.000043	0.00030	0.00018	0.000134601	0.00088	0.00039	0.000457707
Additive Variance		3.35E-05	5.72E-05	0.000044	0.00067	0.00043	0.000630135	0.00077	0.00039	0.000669491
Dominance Variance		1.37E-04	2.58E-04	0.000170	0.00121	0.00071	0.000538405	0.00350	0.00156	0.001830829
Contributions of lines		27.91	16.41	21.53	0.97	1.48	1.38	8.92	16.95	13.65
Contributions of testers		26.16	35.16	32.19	65.30	64.98	65.19	44.70	37.64	40.38
Contributions of lxt		45.93	48.43	46.28	33.73	33.55	33.43	46.38	45.42	45.97

*, **Significant at the P < 0.05 and 0.01 probability level, respectively. ^†^Figures in parentheses indicate individual environment degrees of freedom.

The variance due to hybrids and their interaction with environments (H × E) were highly significant for all characters. However, the contribution of H × E interactions to the variability relative to those due to genetic differences among the hybrids was low for grain Fe content (24%), grain Zn content (23%), grain yield per plant (40%), grain phytate contents (20%), phytate:Fe molar ratio (21%), and phytate:Zn molar ratio (21.4%) and higher for 1000-grain weight (50.3%). Further partitioning of H × E interactions showed that L × E interactions contributed more to the variability relative to line effects, which were 15% for grain Fe content, 19% for grain Zn content, 35% for grain yield per plant, 0.5% for grain phytate contents, 0.2% for phytate:Fe molar ratio, and 0.2% for phytate:Zn molar ratio. However, for thousand grain weight, line effects contributed 42% more to the variability relative to L × E interactions effects. The T × E interaction variance relative to those due to tester effects were 6.4% for grain Fe content, 10% for grain Zn content, 36% for grain yield per plant, 25.4% for grain phytate contents, 4% for phytate:Fe molar ratio, 11.11% for phytate:Zn molar ratio, and 60% for thousand grain weight.

The L × T × E interaction variance relative to that due to L × T interaction was 1% for grain Fe content, 46% for grain Zn content, 53% for grain yield per plant, 26% for grain phytate contents, 80% for phytate:Fe molar ratio, 42% for phytate:Zn molar ratio, and 46% for thousand grain weight. The line × tester interactions contributed more to the variance of test weight than those of lines and testers. The magnitude of σ^2^SCA was quite higher than the magnitude of σ^2^GCA for Fe, Zn, phytate, phytate:Fe/Zn molar ratio, and grain yield.

### 3.2 Parental performance *per se* and combining ability

**Figure 1:**
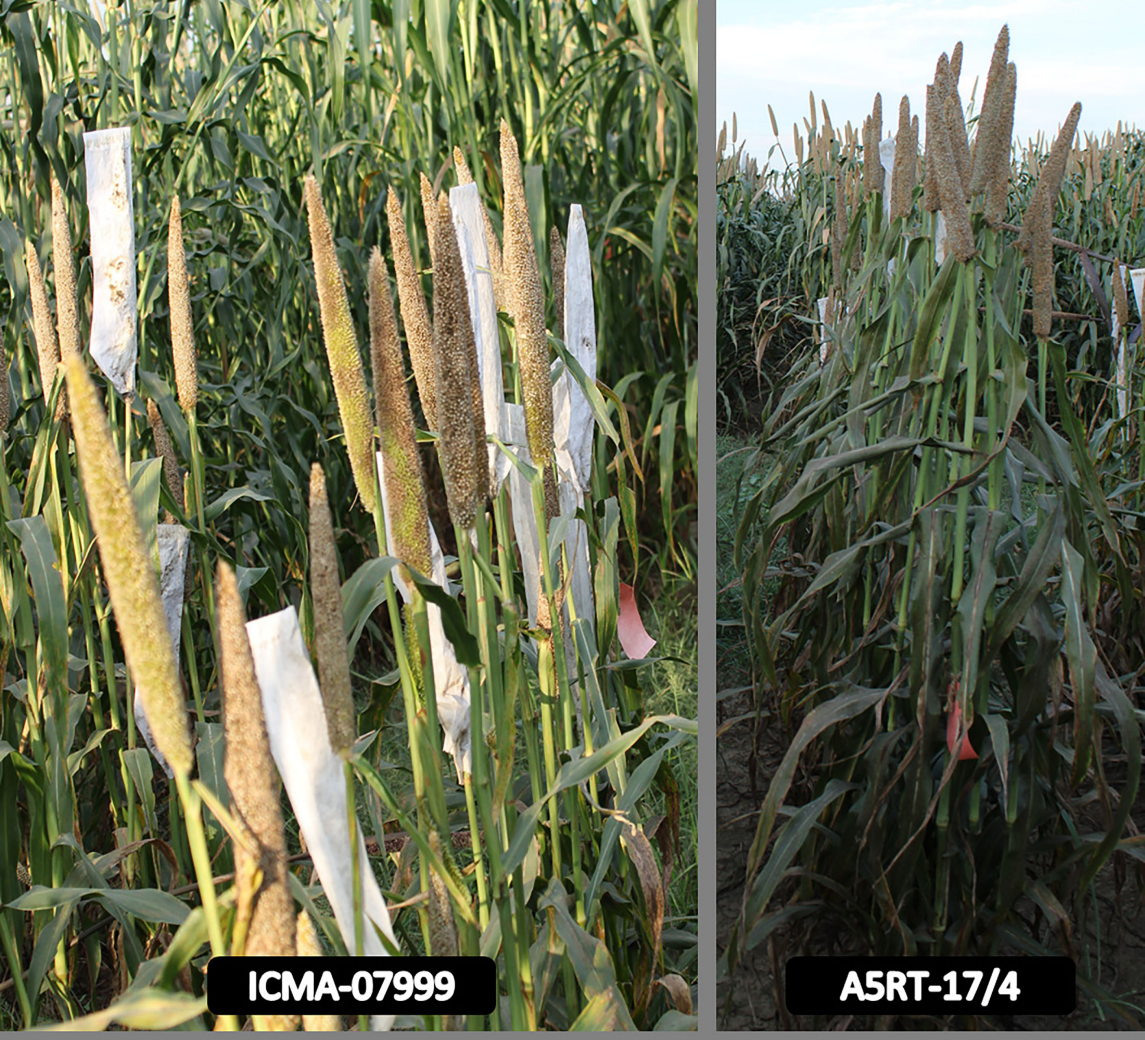
Best general combining parents (line and tester) for both grain Fe and Zn content.

The grain Fe content varied from 49.34 to 66.73 µg/g among the lines and from 39.85 to 65.12 µg/g among the testers (**Table 3**). Two lines (ICMA-07999 and ICMA-12222) showed highly significant GCAs in the positive direction, and one line (ICMA-02555) had significant GCAs in the negative direction for grain Fe content. Similarly, four testers had highly significant GCAs toward positive side, and six testers had significant GCAs for grain Fe content in the negative direction (**Table 3**). Grain Zn content varied between 44.72 and 57.32 µg/g among lines and from 38.38 to 60.86 µg/g among testers. All the three lines had significant positive GCA effects for grain Zn content. Among testers, four testers had highly significant GCAs toward positive side, and six testers had significant negative GCAs for grain Zn content **Figure 1**.

**Table 3:** Performance *per se* of the lines and testers and their general combining ability (GCA) effects for traits studied in pearl millet across two environments.

Parent	Fe (µg g^−1^)		Zn (µg g^−1^)		TGW (g)		GYPP (g)		PA (mg g^−1^)		PA/Fe		PA/Zn	
	Mean (ppm)	GCA	Mean (ppm)	GCA	Mean (ppm)	GCA	Mean (ppm)	GCA	Mean (ppm)	GCA	Mean (ppm)	GCA	Mean (ppm)	GCA
Lines														
ICMA-02555	66.728	−3.39**	57.325	2.1**	11.643	−0.23	48.802	2.74**	0.085	−0.005**	0.112	0.002	0.146	−0.0137**
ICMA-07999	49.343	1.95**	44.715	1.52**	8.983	0.21	68.229	0.550	0.079	0.0046**	0.136	0.001	0.176	0.003
ICMA-12222	51.380	1.43**	51.290	−3.62**	12.275	0.017	58.290	2.19**	0.121	0.000	0.200	−0.0032*	0.235	0.01023**
Testers														
A5RT-17/4	65.125	13.4**	57.078	12.41**	13.905	0.061	43.424	−0.040	0.083	−0.003	0.113	−0.02**	0.145	−0.033**
A5RT-17/5	52.500	−7.63**	51.045	−2.79**	11.564	−0.09	46.337	1.680	0.089	0.0064**	0.145	0.019**	0.174	0.0204**
A5RT-17/6	51.800	−8.07**	44.795	−1.44*	6.455	−0.36	46.526	0.310	0.080	0.005*	0.131	0.017**	0.176	0.0087**
A5RT-17/7	56.068	−10**	53.265	−6.37**	12.570	0.40	43.608	−1.060	0.084	0.000	0.129	0.015**	0.161	0.021**
A5RT-17/8	52.533	−10.9**	49.238	−8.31**	7.397	−0.8**	41.054	0.330	0.091	−0.0046*	0.148	0.01**	0.184	0.018**
A5RT-17/16	39.850	−12.9**	41.328	−6.11**	13.776	0.39	43.900	1.940	0.086	−0.003	0.181	0.015**	0.205	0.006
A5RT-17/19	41.450	−8.9**	38.383	−6.1**	10.742	0.31	54.444	0.160	0.080	−0.0059**	0.163	0.003	0.206	0.002
A5RT-17/25	53.728	15.99**	60.863	7.83**	14.304	−0.42	40.000	−1.150	0.075	−0.001	0.119	−0.0232**	0.129	−0.0221**
A5RT-17/26	54.388	16.72**	59.203	7.49**	9.705	0.17	43.271	−2.340	0.085	−0.001	0.134	−0.0252**	0.150	−0.021**
A5RT-17/29	52.695	12.39**	49.090	3.4**	7.291	0.33	39.454	0.170	0.085	0.0073**	0.139	−0.0112**	0.172	0.000

* and **, significant at the P< 0.05 and 0.01 probability level, respectively.

*Per se* performance in terms of grain yield per plant varied between 48.80 and 68.23 g in lines and from 39.45 to 54.44 g in testers. One line each had highly significant positive and negative GCAs for grain yield per plant. None of the testers had significant positive or negative GCA effects. Thousand grain weight ranged from 8.983 to 12.275 g in lines and from 6.45 to 14.3 g among testers. None of the lines had either significant positive or negative GCA effects. Only one tester had significant GCA effect toward negative side in thousand grain weight.

Grain phytate contents varied from 0.079 to 0.121 mg g^−1^ for lines and from 0.075 to 0.091 mg g^−1^ for testers. Significant negative GCA effect was observed for only one line. Among testers, two testers showed significant GCA effect in the positive direction, and two testers had significant GCA effects in the negative direction. Mean values of bioavailable Fe (phytate:Fe molar ratio) varied between 0.112 and 0.2 among lines and from 0.113 to 0.181 among tester genotypes. Only one line had significant GCA effect in the negative direction for phytate:Fe molar ratio. Five testers had a significant GCA effect toward positive side, and four had significant GCA effects toward negative side for the phytate:Fe molar ratio. For bioavailable Zn (phytate:Zn molar ratio), the mean value varied between 0.146 and 0.235 among lines and from 0.129 to 0.206 in tester genotypes. Among three lines, one had significant GCA effect toward positive side, and one line had significant negative GCA effect. Among testers, four testers had a significant GCA effect in the positive direction, and three testers had significant GCA effect in the negative direction.

### 3.3 Heterosis and combining ability

Grain Fe content for hybrids ranged from 47.9 to 95.6 µg/g over environments. Among all the 30 hybrids, 23 hybrids displayed significant positive MPH, and negative heterosis was observed among the remaining. Similarly, 22 hybrids had significant positive BPH, and five hybrids showed significant negative heterosis. Of the 30 cross-combinations 11 crosses showed a significant SCA effect in the positive direction, and eight crosses had a significant SCA effect in the negative direction. The highest significant positive SCA effect was exhibited by the cross ICMA-02555 × A5RT-17/25 and ICMA-12222 × A5RT-17/16. Similarly, grain Zn content for hybrids ranged from 44.3 to 75.96 µg/g. Among all the 30 hybrids, 15 hybrids showed significant BPH in the positive direction, whereas four hybrids had significant heterosis in the negative direction. Similarly, for MPH, 18 hybrids had significant heterosis in the positive direction, and two hybrids had significant heterosis in the negative direction. Significant positive SCA effect was observed for 12 of the 30 hybrids, whereas significant negative SCA effect for 10 hybrids. The highest significant positive SCA effect was exhibited by the hybrids ICMA-12222 × A5RT-17/5 and ICMA-02555 × A5RT-17/8.

For grain yield per plant, hybrids mean value varied from 43.2 to 54.2 g. Among 30 hybrids, 10 hybrids displayed significant BPH in the negative direction, and no hybrid had significant positive heterosis. Similarly, for MPH, only one hybrid had significant negative heterosis. Of the 30 cross-combinations, none of the crosses displayed a significant positive or negative SCA effect for grain yield per plant. Similarly, thousand grain weight varied from 8.98 to 12.28 g. Twelve of the 30 hybrids showed significant MPH in the positive direction, and none of the hybrids had significant negative heterosis. Similarly, for BPH, only one hybrid had significant negative heterosis, and six hybrids had significant positive heterosis. Of the 30 hybrids, three hybrids showed a significant SCA effect in the positive direction, and four hybrids showed a significant SCA effect in the negative direction. The highest significant positive SCA effect was exhibited by the hybrids ICMA-02555 × A5RT-17/7 and ICMA-12222 × A5RT-17/25.

Averaged over the environments, grain phytate contents varied from 0.076 to 0.11 mg g^−1^. Five of the 30 hybrids displayed significant positive MPH, and two hybrids had significant heterosis in the negative direction. Similarly, 10 hybrids had significant heterosis in the negative direction, and five hybrids had significant BPH in the positive direction. Of the 30 hybrids, five hybrids showed a significant SCA effect toward positive side, and three hybrids showed a significant SCA effect toward negative side. The highest significant SCA effect in the positive direction was exhibited by the hybrids ICMA-07999 × A5RT-17/5 and ICMA-07999 × A5RT-17/26, and the highest significant negative SCA effect was exhibited by the hybrids ICMA-02555 × A5RT-17/25 and ICMA-12222 × A5RT-17/26.

For bioavailable Fe (phytate:Fe molar ratio), the *per se* performance of hybrids varied between 0.071 and 0.148. Among 30 hybrids, 18 hybrids showed significant MPH in the negative direction, and zero hybrids had significant positive BPH. Similarly, 22 hybrids had significant heterosis in the negative direction, and no hybrid had significant positive BPH. Of the 30 hybrids, five hybrids showed a significant SCA effect in the positive direction, and five hybrids showed a significant SCA effect in the negative direction. The highest significant positive SCA effect was exhibited by the hybrids ICMA-02555 × A5RT-17/16 and ICMA-12222 × A5RT-17/25, and the highest significant negative SCA effect was exhibited by the hybrids ICMA-02555 × A5RT-17/25 and ICMA-12222 × A5RT-17/5. Similarly, for bioavailable Zn estimate (phytate:Zn molar ratio), the mean value ranged from 0.11 to 0.22. Among 30 hybrids, 17 hybrids showed significant negative BPH, and only one hybrid had significant positive BPH. Similarly, nine hybrids had significant heterosis in the negative direction, and only one hybrid had significant BPH in the positive direction. Of the 30 hybrids, seven hybrids showed a significant SCA effect toward positive side and seven hybrids showed a significant SCA effect toward negative side. The highest significant positive SCA effect was exhibited by the hybrids ICMA-07999 × A5RT-17/5 and ICMA-12222 × A5RT-17/7, and the highest significant negative SCA effect was exhibited by the hybrids ICMA-12222 × A5RT-17/5 and ICMA-12222 × A5RT-17/8.

**Figure 2:**
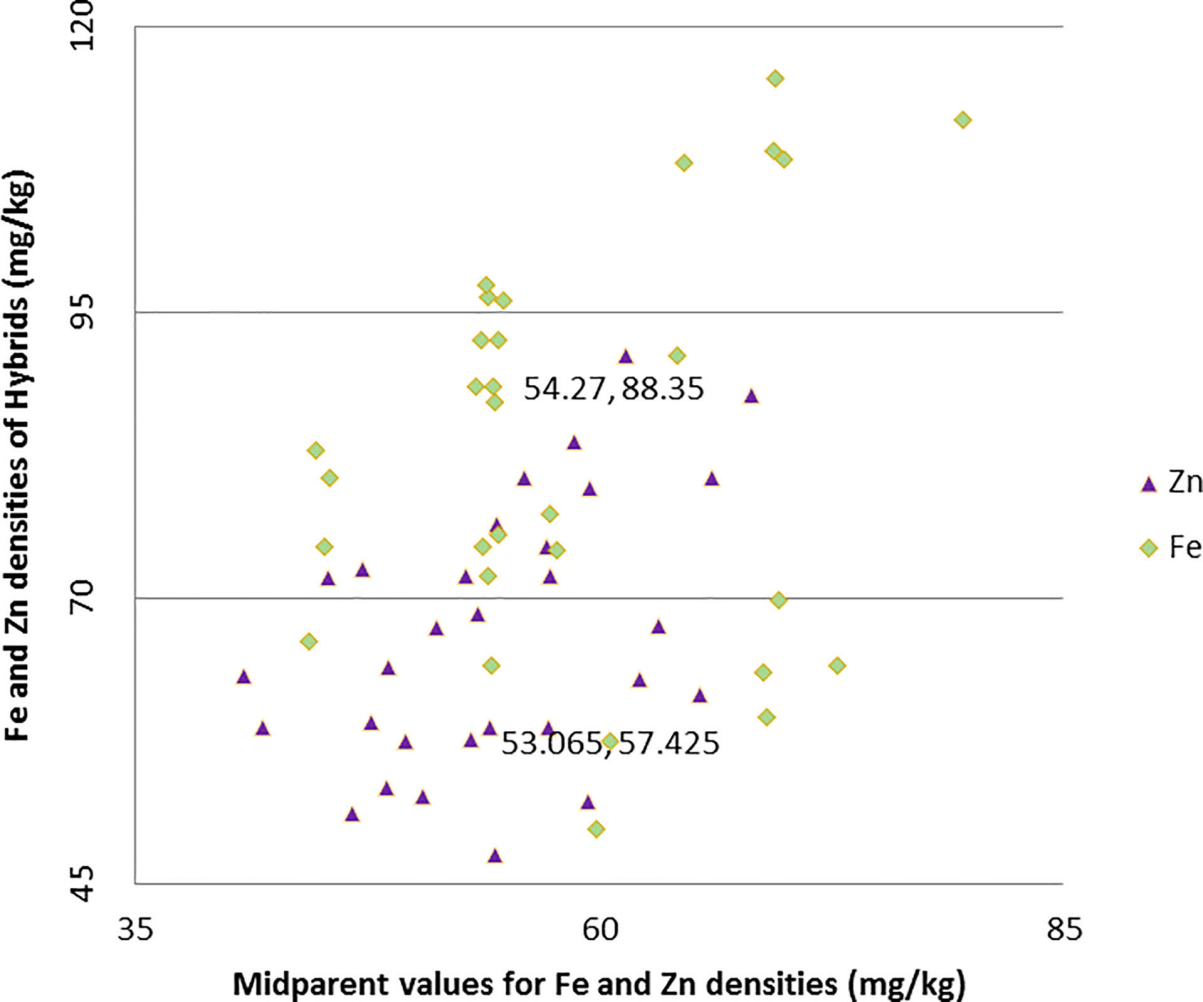
Relationship between grain Fe and Zn densities of hybrids and mid-parent values in line × tester trials across two environments.

**Figure 3:**
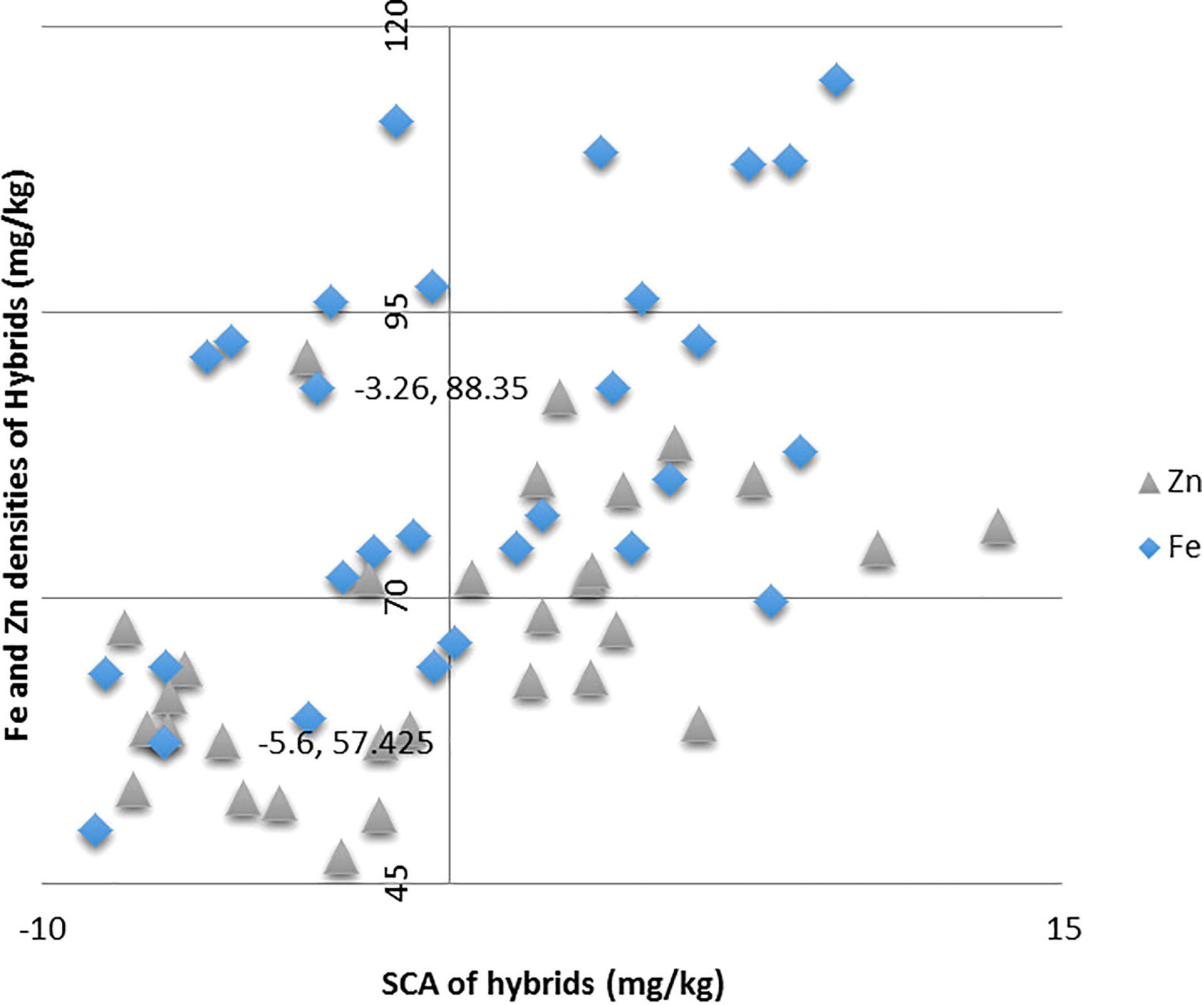
Relationship between grain Fe and Zn densities of hybrids and their specific combining ability (SCA) effects in line × tester across two environments.

Both the hybrids ICMA-02555 × A5RT-17/8 and ICMA-02555 × A5RT-17/8 proved to be the best specific combiner over all the environments for grain Fe and grain Zn content. Similarly, ICMA-12222 × A5RT-17/5 for bioavailable Fe estimate (phytate:Fe molar ratio) and bioavailable Zn estimate (phytate:Zn molar ratio) and ICMA-02555 × A5RT-17/25 is the best specific combiner over all the environments for grain phytate contents. The hybrids, namely, ICMA-02555 × A5RT-17/26 and ICMA-07999 × A5RT-17/25, showed a high *per se* performance, significant heterobeltiosis, SH, and a significant positive SCA effect involving both good and poor GCA. **Table 4** shows the best five hybrids for the pearl millet attributes examined in two environments for performance *per se*, SCA, BPH, and SH. There were significant and negative correlations between performance *per se* of hybrids and mid-parental values for Fe (r = 0.43), and, for Zn, it is positively correlated (r = 0.36 for Zn) across environment (**Figure 2**). Similarly, a highly significant positive correlation was observed between performance *per se* of the hybrids, and their SCA was observed for both Fe density (r = 0.46) and Zn density (r = 0.52) across environment (**Figure 3**).

**Table 4:** Performance *per se*, specific combining ability (SCA), better-parent heterosis (BPH) and standard heterosis (SH) of five top rank hybrids for the traits studied of pearl millet across two environments.

Trait	Hybrid	Performance *per se*			SCA	BPH	CC Pusa 1201
		F1 (hybrid)	P1 (line)	P2 (tester)			
Fe (µg g^−1^)	ICMA-02555 × A5RT-17/25	95.025	66.728	53.728	9.46**	42.41 **	125.55 **
	ICMA-12222 × A5RT-17/16	70.070	51.380	39.850	8.58**	36.38 **	66.32 **
	ICMA-02555 × A5RT-17/26	94.600	66.728	54.388	8.3**	41.77 **	124.54 **
	ICMA-02555 × A5RT-17/8	66.475	66.728	52.533	7.88**	−0.38	57.79 **
	ICMA-07999 × A5RT-17/16	62.113	49.343	39.850	0.103	25.88 **	47.43 **
Zn (µgg^-1^)	ICMA-12222 × A5RT-17/5	65.965	51.290	51.045	13.43**	28.61 **	96.44 **
	ICMA-02555 × A5RT-17/8	63.208	57.325	49.238	10.47**	10.26 *	88.23 **
	ICMA-02555 × A5RT-17/26	75.968	57.325	59.203	7.41**	28.32 **	126.23 **
	ICMA-12222 × A5RT-17/16	55.290	51.290	41.328	6.075**	7.8	64.65 **
	ICMA-12222 × A5RT-17/4	73.225	51.290	57.078	5.49**	28.29 **	118.06 **
TGW (g)	ICMA-02555 × A5RT-17/7	15.982	11.643	12.570	2.95**	27.14 **	7.84
	ICMA-12222 × A5RT-17/25	13.858	12.275	14.304	1.4**	−3.12	−6.49
	ICMA-07999 × A5RT-17/8	13.623	8.983	7.397	1.35**	51.66 **	−8.07
	ICMA-02555 × A5RT-17/5	13.421	11.643	11.564	0.410	15.27	−9.44
	ICMA-12222 × A5RT-17/8	12.899	12.275	7.397	0.820	5.08	−12.96
GYPP (g)	ICMA-02555 × A5RT-17/16	52.075	48.802	43.900	3.641	6.7	−4.46
	ICMA-12222 × A5RT-17/29	54.160	58.290	39.454	2.557	−7.085	−0.64
	ICMA-07999 × A5RT-17/6	52.476	68.229	46.526	2.37	−23.08 **	−3.73
	ICMA-02555 × A5RT-17/7	47.730	48.802	43.608	2.29	−2.19	−12.43
	ICMA-12222 × A5RT-17/4	53.457	58.290	43.424	2.065	−8.29	−1.93
PA (mg g−^1^)	ICMA-07999 × A5RT-17/4	0.083	0.079	0.083	−0.0103**	−0.12	−8.08
	ICMA-02555 × A5RT-17/25	0.076	0.085	0.075	−0.00975**	−10.8	−16
	ICMA-12222 × A5RT-17/26	0.082	0.121	0.085	−0.0091**	−32.89 **	−9.44
	ICMA-07999 × A5RT-17/8	0.085	0.079	0.091	−0.004	−6.45	−5.75
	ICMA-12222 × A5RT-17/5	0.093	0.121	0.089	0.001	−23.5 **	3.22
PA/Fe	ICMA-02555 × A5RT-17/25	0.071	0.112	0.119	−0.02**	−40.18 **	−32.47 **
	ICMA-12222 × A5RT-17/5	0.11	0.200	0.145	−0.017**	−44.86 **	5.21
	ICMA-02555 × A5RT-17/8	0.12	0.112	0.148	−0.01133*	−24.05 **	7.05
	ICMA-12222 × A5RT-17/16	0.114	0.200	0.181	−0.017**	−43.14 **	8.481
	ICMA-02555 × A5RT-17/26	0.08	0.112	0.134	−0.01*	−40.99 **	−24.52 *
PA/Zn	ICMA-12222 × A5RT-17/5	0.144	0.235	0.174	−0.048**	−38.91 **	24.57
	ICMA-02555 × A5RT-17/8	0.15	0.146	0.184	−0.0227**	−22.79 *	23.57
	ICMA-07999 × A5RT-17/7	0.17	0.176	0.161	−0.015*	−3.23	47.82 **
	ICMA-07999 × A5RT-17/4	0.12	0.176	0.145	−0.015*	−34.31 **	0.34
	ICMA-02555 × A5RT-17/7	0.154	0.146	0.161	−0.014*	−4.34	33.64 *

* and **, significant at the P< 0.05 and 0.01 probability level, respectively.

## 4 Discussion

The combining ability analysis revealed the presence of genetic variation among parents and hybrids for the traits studied. Substantial variability was observed for the Fe and Zn content among parents, which has also been reported earlier (Velu et al., 2007; Rai et al., 2012; Govindraj et al., 2013; Kanatti et al., 2014). In comparison with the individual contributing effects of lines and testers, the influence of line tester hybrid was more evident. For the traits studied, the variability among the hybrids due to SCA (σ^2^ SCA) was higher than the variability owing to GCA (σ^2^ GCA) with the predictability ratio being lesser than unity. This revealed that both Fe and Zn grain content is mostly non-additive genetic control as well as the presence of superior crosses with significant SCA and higher heterosis (Nandaniya et al., 2016; Jeeterwal et al., 2017; Warrier et al., 2020). As a result, the material used in this study is best suited for use in the hybrid breeding program. Several earlier studies have reported the predominance of additive variance for grain Fe and Zn in pearl millet (Velu et al., 2011; Govindraj et al., 2013; Kanatti et al., 2014), maize (Arnold and Bauman, 1976; Long et al., 2004; Chen et al., 2007) and rice (Zhang et al., 2004). Both SCA (σ^2^ SCA) and GCA (σ^2^ GCA) were significant with higher σ^2^ SCA for GYPP, demonstrating the significance of both variances with a predominance of non-additive variance. Earlier Nandaniya et al. (2016) and Chittora and Patel (2016) revealed the significance of non-additive gene action for GYPP. According to Kathale et al. (2013) and Ansodariya et al. (2013), the relevance of both additive and non-additive gene action for 1,000-grain weight is significant.

Govindaraj et al. (2013) also observed substantial GCA in both directions for Fe and Zn grain densities. This clearly indicates that gene combinations in different lines were not similar. Likewise, the lines with significant negative GCA for Zn had an equally significant positive GCA for Fe content, and the testers with negative significant GCA for Zn had an equally significant negative GCA for grain Fe content. This showed that the mechanism of Fe and Zn filling in grain in testers is regulated by the same gene(s) or that the variability of both Fe and Zn might be controlled by a close genetic linkage (Kanatti et al., 2014)

This research also identified restorer lines (R) and maintainer lines (B) with a high–grain Fe and Zn content, which are also good general combiners. Furthermore, to be economically viable, genetic enhancement of grain Fe and Zn content must not compromise on grain yield. An earlier research of pearl millet found no negative relationship between these micronutrients and grain output (Gupta et al., 2009). However, in a later study comprising four different hybrid trials, grain Fe content was significantly negatively linked with grain production in two trials and uncorrelated in other two trials. In any of these four trials, Zn density did not correlate with grain yield (Rai et al., 2012). For better clarity, such character association studies must obviously be conducted employing a wide range of materials and diverse environments.

The molar ratio of grain phytate:Fe to Zn and grain phytate content should have negative heterosis to increase the bioavailability of both Fe and Zn. Several parents had non-significant GCA effects for grain phytate, indicating that this trait has dominant genetic control and may be used to reduce grain phytate content. Few hybrids had lower mid-parent values for grain phytate and phytate:Fe/Zn molar ratios. These results indicate that these hybrids have lower levels of these traits with increased bioavailability of Fe and Zn. This finding is consistent with the findings of Brkic et al (2003), who reported some heterotic phytate-lowering effects in maize.

On the basis of the findings, it is possible to infer that there is great genetic potential to increase the bioavailability of grain Fe/Zn content in the lines to provide an inexpensive and long-term solution to the iron-deficient population. Common and overlapping quantitative trait loci (QTL) for Fe and Zn grain density have been identified in wheat (Peleg et al., 2009; Singh et al., 2010), pearl millet (Kumar, 2011), rice (Stangoulis et al., 2007), and beans (Cichy et al., 2009; Blair et al., 2011). Diverse sources of pearl millet with high Fe and Zn grain density levels have been identified, providing valuable experimental materials for mapping population and identifying diverse physiological processes for optimal Fe and Zn accumulation in the grains. Using marker-assisted selection to introgress high Fe and Zn QTL and pyramiding them in parental lines may be the most effective strategy for breeding hybrids with a high Fe and Zn content in pearl millet. The combining ability tests and the manifestation of heterosis in F_1_ provided various cross-combinations that may be advised to enhance the population’s Fe/Zn profile. A plant population with higher grain Fe/Zn content, enhanced Fe/Zn bioavailability, higher grain yields, and lower phytate concentration can be created by delaying the selection in cross-combinations until later generations.

## Data availability statement

The original contributions presented in the study are included in the article/supplementary material. Further inquiries can be directed to the corresponding authors.

## Author contributions

TR and SS planned and designed the research. SS, TR, MS, and MM performed the field experiment. AS and MM helped in Fe and Zn estimation. TR, TS, and MM conducted recording and compilation of data. TR and MS conducted statistical analysis. TR and JM prepared the manuscript. CS edited and revised the manuscript for publication. All authors contributed to the article and approved the submitted version.
